# Hypomethylation and increased expression of the putative oncogene *ELMO3* are associated with lung cancer development and metastases formation

**DOI:** 10.18632/oncoscience.42

**Published:** 2014-05-23

**Authors:** Signe Søes, Iben Lyster Daugaard, Brita Singers Sørensen, Andreas Carus, Manuel Mattheisen, Jan Alsner, Jens Overgaard, Henrik Hager, Lise Lotte Hansen, Lasse Sommer Kristensen

**Affiliations:** ^1^ Department of Biomedicine, University of Aarhus, Aarhus C, Denmark; ^2^ Department of Experimental Clinical Oncology, Aarhus University Hospital, Aarhus C, Denmark; ^3^ Department of Pathology, Aarhus University Hospital, Aarhus C, Denmark; ^4^ Department of Hematology, Rigshospitalet, Copenhagen, Denmark

**Keywords:** Non-Small Cell Lung Cancer, metastases, DNA methylation, oncogenes, microRNA

## Abstract

Numerous genetic and epigenetic events driving tumorigenesis have been characterized. However, knowledge is lacking on the particular events required for the metastatic spread of cancer cells. The engulfment and cell motility 3 (*ELMO3*) gene plays an important role for the migratory potential of cells, but have not previously been studied in primary samples from cancer patients. We collected material from primary non-small cell lung cancer (NSCLC) tumors and paired brain or adrenal gland metastases from 26 patients and from 26 primary tumor samples from metastasis-free patients matched for age, gender, histology, T-stage, smoking status, and proportion of tumor cells. Using reverse transcriptase–quantitative PCR (RT-qPCR) *ELMO3* was shown to be overexpressed in primary tumors from patients with distant metastases compared to normal lung tissue (*p*<0.001), and compared to primary tumors from metastasis-free patients (*p*<0.001). The increased expression coincided with decreased methylation levels of the *ELMO3* promoter region. High expression and hypomethylation of *ELMO3* were also observed when studying the paired brain and adrenal gland metastases. In conclusion, the putative oncogene, *ELMO3*, is overexpressed in NSCLC in combination with hypomethylation of its promoter and these cancer-specific events are associated with the formation of metastases.

## INTRODUCTION

Non-small cell lung cancer (NSCLC) is one of the leading causes of cancer related deaths worldwide [1]. NSCLC progresses, like most other cancers, as a consequence of both genetic and epigenetic changes. As can be observed for most solid cancers, the main cause of death is metastatic spread of the cancer rather than the primary tumor itself. Genome-wide studies of DNA methylation patterns have revealed that aberrations of the methylome can be observed both during initiation and progression of cancer [2, 3]. In lung cancer numerous tumor suppressor genes have been shown to undergo DNA methylation mediated silencing [4]. On the other hand, examples of proto-oncogenes becoming overexpressed as a result of DNA demethylation are sparse. In addition to genetic and epigenetic lesions, numerous microRNAs (miRs) have been shown to be up- or down-regulated in cancer [5], and are promising as diagnostic and prognostic biomarkers [6].

In mammals the engulfment and cell motility (ELMO) family consists of three proteins, ELMO1, ELMO2, and ELMO3. While ELMO2 and 3 are less well characterized, the ELMO1 protein has been shown to play important roles in cytoskeleton rearrangements during phagocytosis and cellular migration [7]. However, ELMO2 and 3 are expected to possess similar functions as ELMO1 [8], and ELMO3 is likely to play important roles in the cell renewing and migration processes within the intestinal epithelia and in colorectal cancer [8]. While it has been found that binding of the transcription factors CDX2 and SP1 to the *ELMO3* promoter activates the gene [8], DNA methylation studies of the *ELMO3* promoter have not been undertaken previously. Furthermore, *ELMO3* expression has not previously been analyzed in primary samples from cancer patients.

The miR-328 gene is located within the same locus as the *ELMO3* gene on chromosome 16q22.1, and it has been indicated that miR-328 may play a role in the migration of NSCLC cells and could potentially be used as a biomarker to differentiate NSCLC patients with brain metastases from patients without brain metastases [9]. In addition, miR-328 expression in whole peripheral blood cells has been proposed as a potential diagnostic biomarker of NSCLC [10].

In this study, we hypothesized that *ELMO3* plays a role in the formation of metastases in NSCLC. We further hypothesized that the expression of *ELMO3* is influenced by methylation of its promoter CpG island. Since overexpression of miR-328 has been observed in NSCLC and this microRNA is located in the same locus as *ELMO3* we investigated whether expression of *ELMO3* is associated with miR-328 expression. Using reverse transcriptase–quantitative PCR (RT-qPCR) and methylation sensitive–high resolution melting (MS-HRM) expression and DNA methylation, respectively, were studied in primary NSCLC tumors and paired brain or adrenal gland metastases from 26 patients as well as in an additional cohort of 26 primary tumor samples from 26 metastasis-free patients matched for age, gender, histology, T-stage, smoking status, and proportion of tumor cells.

## RESULTS

### Expression levels of *ELMO3* in NSCLC

**Figure 1:**
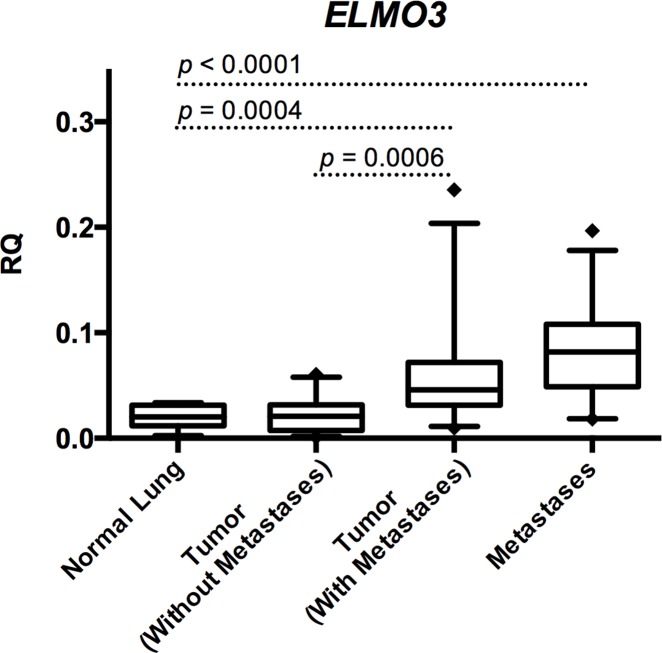
*ELMO3* expression in NSCLC The relative quantitative (RQ) expression of *ELMO3* is on the y-axis. The samples have been divided in four groups: Normal lung, primary tumors from patients without metastases, primary tumors from patients with metastases, and the paired brain or adrenal gland metastases. *P*-values indicating differences between the groups are shown (Students t-test).

*ELMO3* was significantly higher expressed in primary tumors from patients with distant metastases compared to normal lung tissue (*p*=0.0004) (Figure 1). When comparing *ELMO3* expression between the patient cohort without metastases and the cohort with brain- or adrenal gland metastases, it was observed that the expression was significantly higher in the cohort with metastases (*p*=0.0006) (Figure 1). In addition, the expression of *ELMO3* was significantly higher in metastases compared to normal lung tissue (*p*<0.0001) (Figure 1).

### Methylation of *ELMO3* in NSCLC

**Figure 2:**
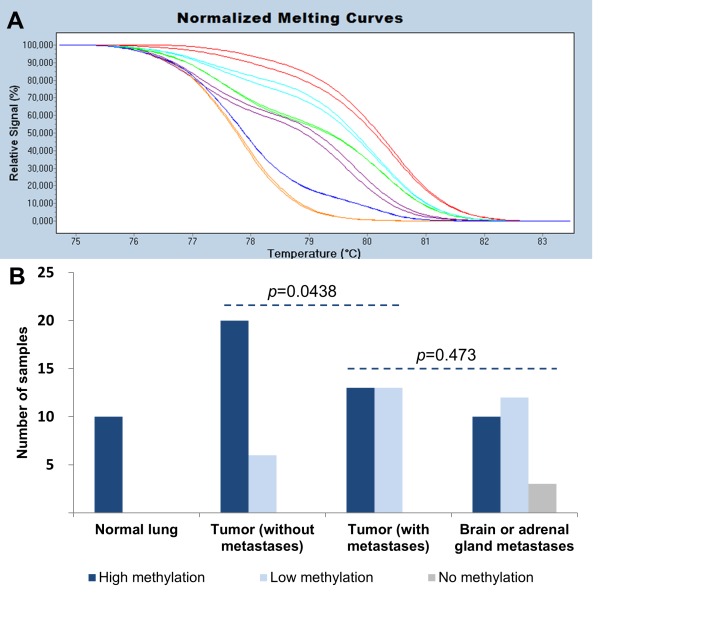
Methylation analyses of *ELMO3* in NSCLC A. Normalized melting curves for the *ELMO3* MS-HRM assay. A primary tumor sample is shown in purple and the matched brain metastasis in turquoise. The standard dilution series of methylated DNA into unmethylated DNA are shown together with the patient samples. The 100% methylated standards shown in red, the 10% methylated standards in green, the 1% methylated standards in blue, and the 0% methylated standards in orange. Samples were run in duplicates. B. All samples were classified into three groups; high methylation (10-100%), low methylation (1-10%), and no methylation (<1%). The number of samples is shown on the y-axis. The four sample groups are shown on the x-axis; normal lung, primary tumors without metastases, primary tumors with metastases, and paired brain or adrenal gland metastases. *P*-values indicating differences between the different groups are shown (χ^2^ test).

MS-HRM was used to semi-quantitatively assess methylation levels of the *ELMO3* promoter region. Melting curves for two patient samples together with standards of known methylation levels are shown in Figure 2A. *ELMO3* was found to be highly methylated in all of the normal lung tissue samples, while significantly lower methylation levels were observed when analyzing the primary tumor samples (both patient cohorts combined versus normal lung tissue, *p*=0.022). When comparing the two patient cohorts it was observed that *ELMO3* was methylated at significantly lower levels in the patient cohort with metastases compared to the patient cohort without metastases (*p*=0.044) (Figure 2B). The methylation levels of the matched metastases were associated with the methylation levels in the primary tumor samples (*p*=0.007), and overall there was no significant difference in methylation levels between the primary tumor samples and the matched metastases (*p*=0.473).

### Methylation of *ELMO3* correlates with expression of *ELMO3* in NSCLC

**Figure 3:**
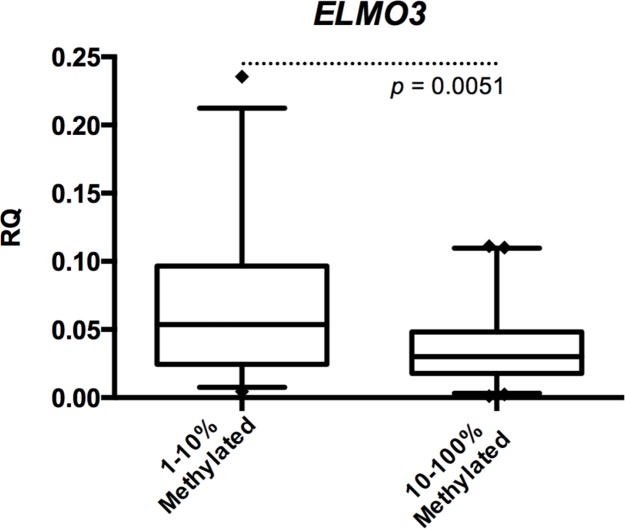
Correlation between methylation and expression of *ELMO3* in NSCLC Comparison of gene expression of *ELMO3* between samples being methylated at high levels (10-100%) and low levels (1-10%). A significantly lower expression of *ELMO3* was observed for the samples having high methylation levels (Students t-test).

When analyzing all samples combined, we found *ELMO3* promoter methylation to be inversely correlated with *ELMO3* expression (*p*=0.005) (Figure 3). We only compared samples methylated at high levels (10-100% methylation) and samples methylated at low levels (1-10% methylation) as the number of samples being unmethylated was too low for meaningful comparisons.

### Expression levels of miR-328 in NSCLC

**Figure 4:**
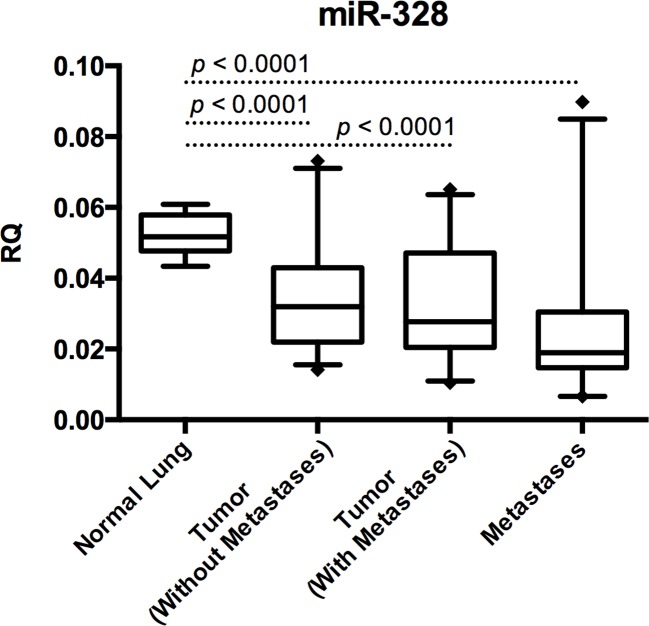
Expression levels of miR-328 in NSCLC Relative quantities (RQ) values for miR-328 expression are shown on the y-axis. The samples have been divided in four groups; normal lung, tumor without metastases, tumor with metastases, and paired brain or adrenal gland metastases. It can be observed that miR-328 was significantly lower expressed in cancerous tissues. *P*-values indicating differences between the different groups are shown (Students t-test).

The expression of miR-328 was found to be significantly lower in primary tumor samples compared to normal lung tissue samples (*p*<0.0001) (Figure 4). However, no difference in expression of miR-328 was observed between the two patient cohorts, as well as between primary tumors and metastases (Figure 4b). The expression of miR-328 was also significantly lower in metastases samples compared to normal lung tissue samples (*p*<0.0001) (Figure 4).

### Inverse correlation between *ELMO3* expression and miR-328 expression

**Figure 5:**
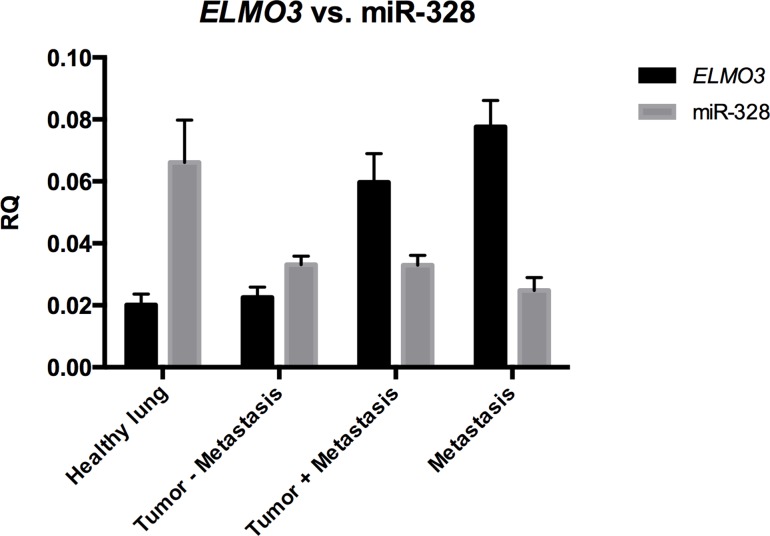
Expression of miR-328 and *ELMO3* in NSCLC Relative quantities (RQ) values for both miR-328 (grey) and *ELMO3* (black) are shown on the y-axis. Samples were divided in four groups; normal lung, tumor without metastases, tumor with metastasis, and paired brain or adrenal gland metastases. It can be observed that *ELMO3* expression was higher in primary tumors from patients with metastases and the paired metastases compared to normal lung, while the opposite is observed for miR-328.

**Figure 6:**
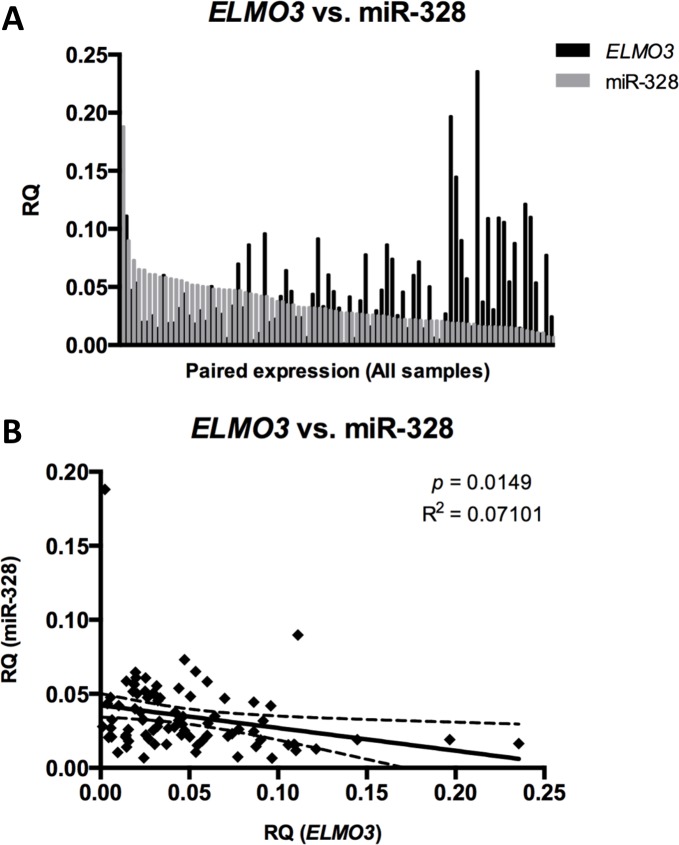
Expression of miR-328 and *ELMO3* in individual samples Comparison of Relative quantities (RQ) values between *ELMO3* and miR-328. A. The paired expression for *ELMO3* (black) and miR-328 (grey) plotted for each sample. Expression is on the y-axis and samples on the x-axis. B. miR-328 expression (y-axis) plotted against *ELMO3* expression (x-axis).

Because miR-328 is located within the same locus as *ELMO3*, it was evaluated whether *ELMO3* - and miR-328 expression was correlated. Initially, we found that *ELMO3* expression was generally low in the normal lung samples, which had a relatively high miR-328 expression, while the opposite was observed for the metastases samples (Figure 5). The miR-328 - and *ELMO3* expression patterns were also analyzed in individual samples, by plotting miR-328 expression as a function of *ELMO3* expression (Figure 6A and 6B). These analyses revealed that that *ELMO3* - and miR-328 expression were inversely correlated.

## DISCUSSION

The presence of distant metastases has a negative impact on the survival of cancer patients, and often causes additional morbidities. In particular, brain metastases may lead to loss of motor and sensory function, and cognitive decline [11]. In NSCLC 10-25% of the patients have already developed brain metastases at the time of diagnosis, while 40-50% will develop them during progression of their disease [12]. Furthermore, brain metastases are difficult to treat successfully as the blood-brain barrier prevents most drugs from reaching and killing the metastatic cells. In order to develop better treatment and prevention strategies it is important to achieve a better understanding of the molecular mechanisms employed by the cancer cells to gain the ability to survive in the blood stream and eventually colonize distant organs, such as the brain and adrenal glands.

Animal models and cells in culture may provide experimental systems for studying the molecular mechanisms behind the development of metastases. However, it has been argued that a comparison of primary tumor tissue with a surgically resected brain metastasis from the same patient provides the best evidence for gene expression changes associated with the metastatic process [11]. We further rationalized that it may be difficult to distinguish passenger events from true metastatic drivers when comparing primary tumors and metastases as a particular metastasis may have been founded by a single clone or only a few cells, which have a background of genetic and epigenetic alterations that may differ from the major clone within the primary tumor. To circumvent this potential problem we decided to assemble an additional patient cohort of 26 metastasis-free lung cancer patients matched for age, gender, histology, T-stage, smoking status, and proportion of tumor cells. The matched cohort had at least five years of recurrence-free survival, which makes the presence of occult metastasis unlikely. Hence, molecular alterations between normal lung tissue and tumor tissue, which are specific to the cohort of patients, who had developed distant metastases, and also found in the paired metastases, are likely to be true drivers of the metastatic process.

*ELMO3* has not previously been described as being implicated in lung cancer development, and studies of its expression or regulation in primary patient samples have not previously been reported. We chose to study this gene because its closely related family member *ELMO1* encodes a protein, which plays important roles in cytoskeleton rearrangements during cellular migration [7]. Using a gene specific RT-qPCR assay and carefully chosen reference genes for normalization purposes [13], we have shown that *ELMO3* overexpression is likely to promote the ability of lung tumor cells to migrate through the blood stream and colonize distant organs. The overexpression of *ELMO3* observed in primary tumors and paired brain or adrenal gland metastases coincided with hypomethylation of its promoter CpG island. This is in agreement with the well-established concept that promoter methylation is normally associated with gene silencing [14]. Furthermore, we observed a highly significant correlation between the methylation levels within primary tumors and matched metastases, making it likely that this molecular aberration is gained by the metastatic cells before colonization of the distant sites.

Interestingly, miR-328, which has previously been associated with the development of NSCLC brain metastases [9], is located within the same locus as *ELMO3*. Therefore, we decided to investigate if *ELMO3* expression and miR-328 expression are correlated. We were surprised to see that miR-328 was down-regulated in cancerous tissues compared to normal lung tissue, since Arora and colleagues found this miR to be overexpressed in the primary tumors from a cohort of patients with brain metastases compared to a matched cohort without brain metastases [9]. However, their study was limited to only a few patients, and did not include comparisons to normal lung tissue. Furthermore, only a single reference gene (5S-rRNA) was used for normalization of RT-qPCR data, and this reference was the least stable reference in a study aiming at identifying stable reference RNA targets for RT-qPCR analyses of normal and cancerous human solid tissues, including lung cancer [15]. Another study found that miR-328 acts as a tumor suppressor in glioblastoma, and patients with low miR-328 expression have poor survival [16]. In our study there was no difference in miR-328 expression between the patient cohorts with and without distant metastases. Thus, our results do not support the use of miR-328 as a biomarker for stratifying NSCLC patients at higher risk of developing brain metastases.

Moreover, we found a weak but statistically significant negative correlation between *ELMO3* expression and miR-328 expression. This may, in part, be explained by miR-328 and *ELMO3* being transcribed in opposite directions, and though miR-328 is located within the *ELMO3* locus it is not expected to be a splicing-derived miR (mirtron) [17]. An obvious explanation could also be that *ELMO3* mRNA is a target for miR-328. However, several *in silico* tools for target prediction (TargetScan, PicTar, MiRanda) did not predict *ELMO3* to be a miR-328 target.

In this study we did not evaluate whether *ELMO3* expression or promoter methylation could be used as a prognostic or predictive biomarker, but it could certainly be interesting to study *ELMO3* further in prospective cohorts to investigate its clinical potential alone or in combination with other biomarkers. In addition, it could be interesting to investigate if *ELMO3* behaves as an oncogene in animal and cellular experimental models to further illuminate its role as a metastases promoting oncogene. Since only few examples of oncogenes being overexpressed as a result of promoter hypomethylation are known, and *ELMO3* methylation has not previously been studied, it could also be of interest to study *ELMO3* promoter methylation and expression in other malignancies.

In conclusion, the putative oncogene, *ELMO3*, becomes overexpressed in NSCLC in combination with DNA hypomethylation of its promoter, and this cancer-specific event is associated with the formation of metastases. Moreover, expression of miR-328, located within the *ELMO3* locus, is inversely correlated with the expression of *ELMO3*. Future research may elucidate if hypomethylation and overexpression of *ELMO3* are implicated in other malignancies as well, and whether ELMO3 could be a potential drug target for cancer treatment.

## MATERIAL AND METHODS

### Patient samples

Seventy-eight formalin-fixed, paraffin-embedded (FFPE) archival blocks from surgical resections from 52 patients diagnosed with NSCLC were retrieved from the Institute of Pathology, Aarhus University Hospital. The material consisted of two cohorts matched on age, gender, smoking status, T stage, histology, and proportion of tumor cells. The first cohort comprised of 26 primary NSCLC tumors with either paired brain metastases (n=21) or paired adrenal gland metastases (n=4). For one metastasis sample extraction of nucleic acids was unsuccessful. Hence data was only obtained for 25 paired metastases samples. The second cohort comprised of 26 primary NSCLC tumors from patients without distant metastases and at least 5 years of recurrence free survival following surgery. In addition, ten normal lung tissue samples derived from NSCLC patients were used as controls. From each sample 1 × 7 μm sections were cut for RNA/small RNA extraction and 5 × 10 μm sections were cut for DNA extraction.

The study was approved by the regional Ethical Committee (journal number M-20110270).

### RNA extraction and cDNA synthesis

RNA extraction was performed using the Tissue Preparation System together with VERSANT Tissue Preparation Reagents and a fully automated, bead-based RNA isolation method (Siemens Healthcare Diagnostics, Tarrytown, NY, USA). The procedure has previously been described [18]. Potential genomic DNA contamination was removed by DNase I treatment. cDNA was generated by using the High Capacity cDNA RT kit (Applied Biosystems, CA, USA) in accordance with the manufacturer's protocol.

### Reverse transcription quantitative PCR (RT-qPCR) analysis of *ELMO3*

cDNA was preamplified using TaqMan PreAmp kit (Applied Biosystems) according to the manufacturer's protocol with slight modifications; 25 μl of TaqMan PreAmp was mixed with 12 μl of pooled assay mix for all genes (*ELMO3* and reference genes) and 13 μl of cDNA. Before performing the qPCR experiments the pre-amplified cDNA was diluted 1:6 in TE buffer. IDT PrimeTime^™^ Std qPCR assay (Integrated DNA Technologies, Coralville, Iowa, USA) was used. The assay was designed with short amplicon length (98bp) and covering the exon 6 – exon 7 boundary in order to avoid amplification of potential contaminating genomic DNA. *ELMO3* primers and probe were: forward; 5′-GGCCTTCTCAGAGCTCATG-3′ and reverse; 5′-TGAGGTTCATGTTCACGTAGC-3′ Probe: 5′-/56-FAM/AGCATCCCC/Zen/ TTTGTGAGGAAGGTG/3IABkFQ/-3′. The experiment was performed on an ABI PRISM^®^7900HT (Applied Biosystems) in 384 well plates assembled by a Biomek 3000 (Beckman Coulter, Brea, California, USA). The final reaction mixture consisted of 3 μL of pre-amplified cDNA with 2× TaqMan Genotyping Master Mix (Applied Biosystems) and 1×IDT assay including primers and probes in a final volume of 15 μL. *ELMO3* expression was normalized using the geometric mean of three reference genes, *IPO8*, *PUM1*, and *TBP* (ABI assays Hs00183533_m1, Hs00472881_m1, and Hs00427621_ m1, respectively), which we previously have shown to be stably expressed in NSCLC [13]. Negative and positive controls for reverse transcription and qPCR step were included together with no template control. qPCR was performed in duplicates for each sample.

### Extraction of small RNA

Total RNA, including miRNA, was extracted using miRNeasy FFPE kit (Qiagen, Hilden, Germany) according to the manufacturer's instructions. Deparaffinization was done using xylene, and the elution volume was 17 μL RNase-free water. Potential genomic DNA contamination was removed by DNase treatment. DNA concentrations and purity were assessed using a NanoDrop 1000 spectrophotometer (Thermo Scientific, Waltham, Massachusetts, USA).

### cDNA synthesis and RT-qPCR analysis of miR-328

cDNA synthesis and RT-qPCR were performed using the miRCURY LNA^™^ Universal RT microRNA PCR system (Exiqon, Vedbaek, Denmark). The reverse transcription reaction was carried out according to the manufacturer's protocol using 10 ng RNA as input. cDNA synthesis was performed in duplicate for each sample. The RT-qPCR was performed using ExiLENT SYBR^®^ master mix and LNA^™^ PCR primer sets, UniRT (Exiqon) for hsa-miR-328 and three potential miR reference genes (hsa-miR-191-5p, hsa-miR-103a-3p, hsa-miR-423-5p), of which two previously have been found to be stably expressed in NSCLC [15]. All data were analyzed with geNorm [19] and NormFinder [20] to measure expression stability. Hence, expression data were normalized to the geometric mean of the two most stable references, hsa-miR-103a-3p and hsa-miR-423-5p.

Negative controls included were cDNA (no RNA template) and cDNA (no RT enzyme) and positive controls included were the three potential miR references (hsa-miR-191-5p, hsa-miR-103a-3p, hsa-miR-423-5p).

### DNA extraction

Five tissue sections of 10 μM were transferred into a microcentrifuge tube and DNA was extracted using the QIAamp DNA FFPE Tissue Kit (Qiagen) according to the manufacturer's instructions. DNA concentrations and purity were measured using a Nanodrop 1000 spectrophotometer (Thermo scientific).

### Sodium Bisulfite treatment

For each sample, five hundred nanograms of genomic DNA were subjected to sodium bisulfite treatment with the EZ-96 DNA Methylation-Gold™ kit (Zymo Research, Irvine, California, USA) according to the manufacturer's instructions.

### Methylation-sensitive high-resolution melting (MS-HRM)

Primers were designed to amplify both methylated and unmethylated DNA according to the guidelines given in [21]. *ELMO3* primers were: forward; 5′-TGGTTAGGAGTAGTAGTTTGGGT-3′ and reverse; 5′-AATCCTCCCTTTCCGAAACCTA-3′ giving rise to an amplicon of 67 bp. The assay was optimized using a dilution series of methylated DNA (fully methylated from Zymo Research) into unmethylated DNA, prepared as described previously [22].

PCR cycling and HRM analysis were performed on the Lightcycler^®^ 480 (Roche Applied Science, Mannheim, Germany). The reaction mixtures consisted of 20 ng of bisulfite modified DNA, 0.5 μL of each primer (final concentration of 500 nM), 1.2 μL of MgCl_2_ (final concentration of 3 mmol/L), 1 μL H_2_0 and 4.8 μL of Lightcycler^®^ 480 High Resolution Melting Master Mix (Roche), in a total volume of 10 μL. The cycling protocol started with one cycle of 95°C for 10 minutes followed by 50 cycles of 95°C for 5 second, 61°C for 10 seconds, 72°C for 10 seconds, one cycle of 95°C for 1 minute, one cycle of 65°C for 1 minute, and a melt from 65°C to 95°C with a temperature increase of 0.1°C/sec. All samples were analyzed in duplicates.

### Data analysis

*ELMO3* expression data were analyzed using RealTime Statminer (Integromics, Granada, Spain) and ΔCt values were generated by normalizing to the geometric mean of the three reference genes. Data for miR-328 expression were analyzed using the Lightcycler^®^ 480 software and ΔCt values were generated by normalizing to the geometric mean of the two reference miRs. Relative quantities (RQ) were generated as 2^(-ΔCt). miR-328 expression in each sample was determined as the average of the cDNA synthesis duplicates.

In MS-HRM, the methylation level of each sample was assessed by comparison of the melting profiles between each sample and the standards of known ratios of methylated and unmethylated templates as described in [23], and scored as being methylated at low level (1-10%), high level (10-100%) or as unmethylated (0-1%).

### Statistical analysis

The expression of *ELMO3* and miR-328, respectively, was compared between groups by unpaired Student's t-tests as the data were normally distributed. The correlation between *ELMO3* and miR-328 expression was assessed by Pearson's correlation analysis. Differences in methylation status between the cohorts were determined using a χ^2^ test. Correlation analyses for methylation status among the paired samples were also performed using a χ^2^ test but with a Yates' correction to prevent overestimation of statistical significance for small data sets. Statistical tests were done using GraphPad Prism version 6 software (GraphPad Software, La Jolla, California, USA). Two-tailed *p*-values of 0.05 or less were considered statistically significant.
